# Notes and Notices

**Published:** 1899-03

**Authors:** 


### NOTES AND NOTICES.

Obituary.—Doctors E. M. Hale and T. S. Hoyne, of Chicago; Dr. W. H. Dickinson, of Des Moines, Iowa; Dr. Virgil L. Todd, formerly of Homœopathic Medical College of Kansas City, died of pneumonia, December, 1889.—*Medical Arena,* March, 1899.

Dr. Givens's Sanitarium for Mental and Nervous Diseases and Habitues of Drugs and Stimulants, at Stamford, Conn., offers unexcelled advantages for those requiring special treatment. It is located within 50 minutes of New York City, on a hill overlooking Long Island Sound, and with 42 trains each way, daily.

During the past year another cottage and many improvements have been added, and the place is up to date in every respect.

The Story of The Philippines.—Active solicitors wanted everywhere for *The Story of the Philippines,* by Murat Halstead, commissioned by the government as Official Historian to the War Department. The book was written in army camps at San Francisco, on the Pacific, with General Merritt, in the hospitals at Honolulu, in Hong Kong, in the American trenches at Manila, in the insurgent camps with Aguinaldo, on the deck of the "Olympia" with Dewey, and in the roar of battle at the fall of Manila. Bonanza for agents. Brimful of original pictures taken by government photographers on the spot. Large book. Low prices. Big profits. Freight paid. Credit given. Drop all trashy unofficial war books. Outfit free. Address, H. L. Barber, General Manager, 356 Dearborn Street, Chicago.

The Alumni Association of the Hahnemann Medical College, Philadelphia.—The annual re-union and banquet of the Alumni Association of the Hahnemann Medical College, Philadelphia, will be held on Wednesday, May 10th, 1899.

The business meeting will convene at 4.30 P. M. in Alumni Hall, Hahnemann Medical College, Broad Street above Race, Philadelphia, and the banquet will be held at 9.45 P. M. at the Walton, S. E. corner Broad and Locust Streets.

The Trustees and Faculty of the College extend a cordial invitation to all the members of the Alumni and their friends to attend the Fifty-first Annual Commencement, to be held on the same evening, at 8 o'clock, at the Academy of Music, S. W. corner Broad and Locust Streets, Philadelphia.

Banquet cards can be secured by notifying the Secretary. Requests received after Wednesday, May 9th, 1899, cannot be considered.

W. D. Carter, M. D., *Secretary,*

1533 South Fifteenth Street, Philadelphia.

ALUMNI ASSOCIATION OF THE NEW YORK HOMŒOPATHIC MEDICAL COLLEGE.—Thursday, May 4th, is the date set for Alumni Day this year. Dr. Helmuth writes: "A carefully prepared programme of the exercises is now being arranged by the Faculty, and additional care is to be extended over all the named clinics, in order that the day may be one of instruction as well as of social reunion."

The annual meeting is the same evening at half-past six, at Delmonico's, Fifth Avenue and Forty-fourth Street. The banquet follows, and promises to outdo the successes of previous years as an elaborate post-prandial programme has been arranged. The price of the dinner will be $4.00, and all Alumni and friends will be welcome. Send early for tickets to Chas. Helfrich, M. D., 64 West Forty-ninth Street, New York.

EDWIN S. MUNSON, M. D., *Corresponding Secretary,*
16 West Forty-fifth Street, New York.

SEE two girls in another column gathering grapes for Speer's wine. Read all about them. Speer's wines are unexcelled by any in the world. AMERICAN INSTITUTE FARMER'S CLUB report the wines of Alfred Speer, of Passaic, New Jersey, the most reliable, and his Oporto grape wine as superior to any in the world.

SCIENTIFIC AMERICAN.—The most popular scientific paper in the world. Established 1845. Weekly, $3.00 a year. Is the only journal published in this country which is devoted to a general treatment of the development of the sciences, arts, and manufactures. It appeals particularly to the inventor, as it is full of suggestive articles relating to the mechanical progress of the day, and it contains a complete list of the patents issued each week, together with the name of the patentee and the title of the invention. Each issue is embellished with numerous illustrations, showing great engineering works, the most recent inventions in bicycles and motor carriages, new forms of machinery, photography, the latest additions to the navy, new guns, locomotives, etc. Many of our patrons have been on our subscription books for a period of thirty or forty years, and we often receive letters from old readers stating that they owe their success in life more to having had the *Scientific American* as their constant friend and companion than to any other one cause. This journal has probably a larger foreign circulation than any other journal printed in the English language.

MUNN & CO., *Publishers,*
361 Broadway, N. Y.

SEE two girls in another column gathering grapes for Speer's wine. Read all about them. Speer's wines are unexcelled by any in the world. ESPECIALLY FOR DELICATE LADIES AND THE AGED. For medicinal use, physicians say Speer's Port wine surpasses the imported. Delicate ladies and aged people find it the best wine to be procured.

